# Cyclotides Suppress Human T-Lymphocyte Proliferation by an Interleukin 2-Dependent Mechanism

**DOI:** 10.1371/journal.pone.0068016

**Published:** 2013-06-26

**Authors:** Carsten Gründemann, Kathrin Thell, Karin Lengen, Manuel Garcia-Käufer, Yen-Hua Huang, Roman Huber, David J. Craik, Gernot Schabbauer, Christian W. Gruber

**Affiliations:** 1 Center for Physiology and Pharmacology, Medical University of Vienna, Vienna, Austria; 2 Center for Complementary Medicine, Department of Environmental Health Sciences, University Medical Center Freiburg, Freiburg, Germany; 3 Institute for Molecular Bioscience, the University of Queensland, Brisbane, Queensland, Australia; New York University, United States of America

**Keywords:** cyclotide, lymphocytes, proliferation, immunosuppression, plant, peptide, cytokine

## Abstract

Cyclotides are a diverse and abundant group of ribosomally synthesized plant peptides containing a unique cyclic cystine-knotted topology that confers them with remarkable stability. Kalata B1, a representative member of this family of mini-proteins, has been found to inhibit the proliferation of human peripheral blood mononuclear cells. Analysis of T-cell proliferation upon treatment with chemically synthesized kalata B1 mutants revealed a region comprising inter-cysteine loops 1 and 2 of the cyclotide framework to be important for biological activity. Cytokine signaling analysis using an ‘active’ kalata B1 mutant [T20K], and the reference drug cyclosporin A (CsA) demonstrated that treatment of activated T-lymphocytes with these compounds decreased the expression of the interleukin-2 (IL-2) surface receptor as well as IL-2 cytokine secretion and *IL-2* gene expression, whereas the ‘inactive’ kalata B1 mutant [V10K] did not cause any effects. The anti-proliferative activity of [T20K] kalata B1 was antagonized by addition of exogenous IL-2. Furthermore, treatment with [T20K] kalata B1 led to an initial reduction of the effector function, as indicated by the reduced IFN-γ and TNF-α production, but the levels of both cytokines stabilized over time and returned to their normal levels. On the other hand, the degranulation activity remained reduced. This indicated that cyclotides interfere with T-cell polyfunctionality and arrest the proliferation of immune-competent cells through inhibiting IL-2 biology at more than one site. The results open new avenues to utilize native and synthetically-optimized cyclotides for applications in immune-related disorders and as immunosuppressant peptides.

## Introduction

The immune system is in constant balance, detecting and eliminating pathogens and cancer cells, but sparing the body’s own cells and tissues. Immune dysfunction can cause an over-reactivity of this defense machinery and in some instances lead to organ rejection and autoimmune diseases, such as multiple sclerosis or rheumatoid arthritis. Immunosuppression, i.e., the targeted reduction of the activation or efficacy of the immune system, is an option for the treatment of such conditions. Since most immune disorders are characterized by an increased proliferation of T-lymphocytes, most immunosuppressive drugs aim at blocking this step in the cell cycle [1].

Naturally occurring peptides, such as cyclosporin A (CsA), a cyclic non-ribosomal depsipeptide of fungal origin, have long been known as effective immunosuppressant drugs to treat increased T-cell proliferation, e.g., severe cases of rheumatoid arthritis [2,3]. Although still widely used in the clinic today, CsA has many and sometimes strong side effects [4]. Other peptides with potential activity in suppressing immune cell proliferation have been isolated from various organisms, including fungi (e.g., colutellin A [5]), plants (e.g., orbitides [6]) and animals (e.g., the scorpion venom peptides margatoxin [7] and Vm24 [8,9]). Another large and diverse group of naturally occurring peptides that have attracted much attention in pharmaceutical drug design applications due to their unique three-dimensional topology, are plant cyclotides [10]. Recently, their anti-proliferative effects on activated human peripheral blood mononuclear cells (PBMC) were reported [11].

Cyclotides are head-to-tail cyclized peptides in which the circular backbone chain comprises ~30 amino acids, including six conserved cysteines that form three disulfide bonds arranged in a cyclic cystine-knot motif [12]. Their remarkable structure make cyclotides extremely resistant to enzymatic, chemical or thermal degradation [13]. In contrast to non-ribosomally synthesized secondary plant metabolites, cyclotides are true gene products and their biosynthesis involves ribosomal precursor synthesis, enzymatic processing [14,15] and protein folding events [16,17]. Cyclotides were originally discovered in coffee-family (*Rubiaceae*) plants [18,19], but have since been found in violets (*Violaceae*) [20,21], cucurbits (*Cucurbitaceae*) [22], legumes (*Fabaceae*) [23,24], and potato-family (*Solanaceae*) plants [25]. Additionally, acyclic cyclotide homologs have been reported in grasses (*Poaceae*) [26,27]. Interestingly, one plant can produce dozens of different cyclotides [11,28] and approximately 300 cyclotides have been sequenced so far (www.cybase.org.au). The intra-cysteine sequences found in naturally occurring cyclotides display a wide range of residue variations and hence the cyclotide scaffold serves as a natural combinatorial peptide template [29]. Based on their phylogenetic distribution in the plant kingdom, it has been estimated that there are at least 50,000 novel cyclotides to be discovered [19], and we are only beginning to understand their enormous variety in plants [30,31].

Their immunosuppressant activity, natural sequence variability, and their biological and chemical stability as well as reported oral activity [32] make cyclotides ideal candidates for drug design applications [33–36]. Here, structure-activity relationships for several amino acid mutants of the cyclotide kalata B1 have been determined using T-cell proliferation assays. After defining the ‘active’ and ‘inactive’ immunosuppressive motifs of the cyclotide framework, we further explored their mechanism-of-action on immunological signaling pathways that are relevant to the regulation of T-cell proliferation *in vivo*. To our knowledge, this is the first detailed report on the modulation and mechanism of cyclotides on signaling pathways in immune cells. The results may open new avenues for applications of cyclotides in immune-related disorders and their *in vivo* analysis in animal models.

## Results and Discussion

The aim of this study was to elucidate immunosuppressive structure-activity relationships of representative cyclotides and to characterize their mechanism-of-action by modulation and analysis of specific immunological signaling pathways that are involved in the physiological regulation of T-lymphocyte proliferation.

### Peptide synthesis and structural analysis

**Figure 1:**
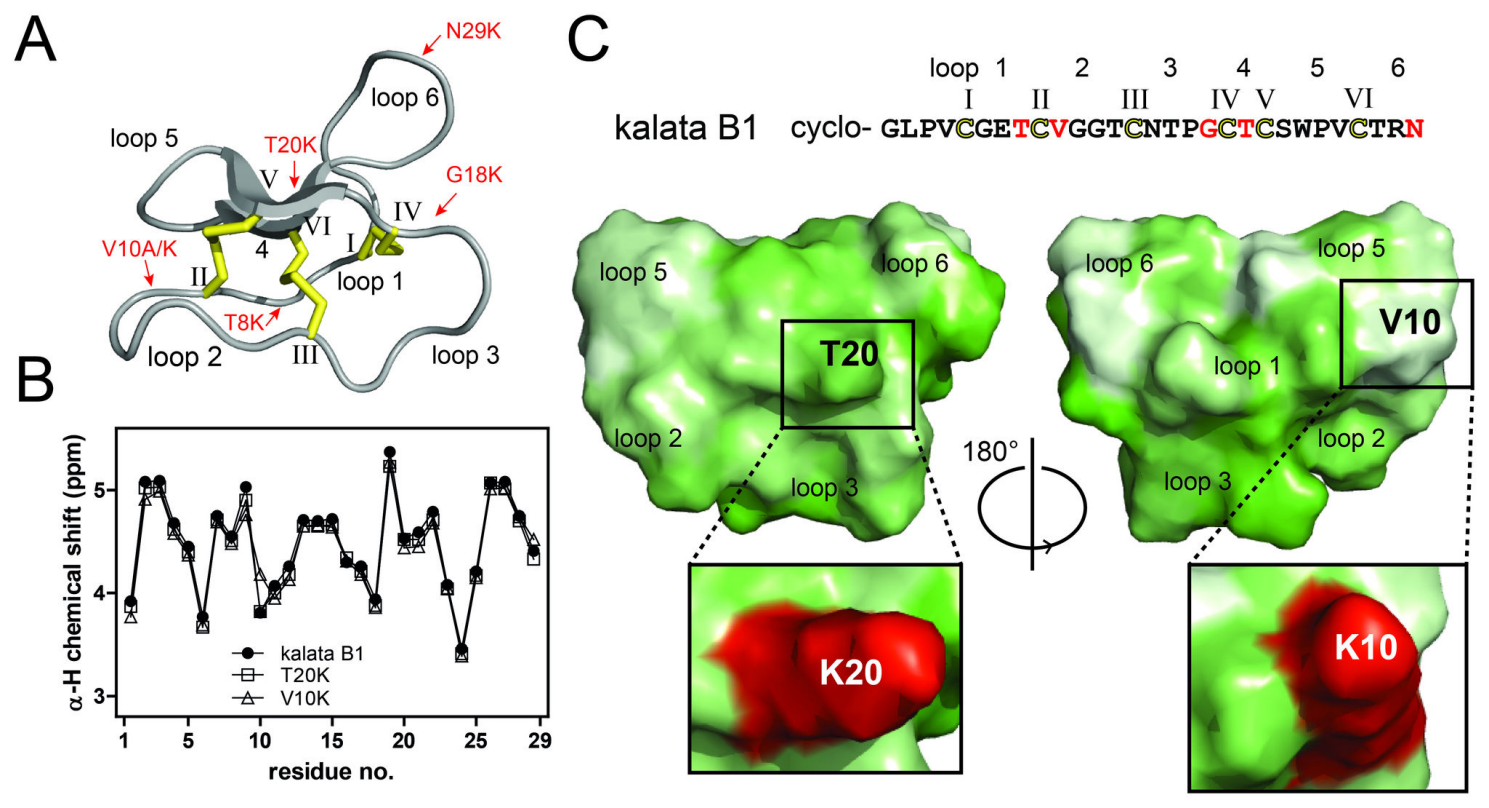
Structure of kalata B1 and cyclotide mutants. The structure of kalata B1 is shown in cartoon form (**A**). The six conserved cysteines are labeled with Roman numerals and the cystine knot disulfide connectivity (C_I_-C_IV_, C_II_-C_V_ and C_III_-C_VI_) is indicated. The amino acid sequence and the loops of the kalata B1 backbone are shown in C. The positions of the mutations are indicated by red arrows in the cartoon and are highlighted with red letters in the sequence. The α-H chemical shift comparison of kalata B1 (full circles), [V10K] kalata B1 (open triangles) and [T20K] kalata B1 (open squares) is shown for all residues starting from residue G1 (**B**). The amino acid sequence (with mutations highlighted in red) and a surface representation of kalata B1 (PDB code: 1NB1) is shown in (**C**). The structure is using a hydrophobicity scale according to Eisenberg et al. [53] to illustrate the amphiphilic nature of these peptides. Typical hydrophilic regions of cyclotides are in loops 2 and 3, whereas the hydrophobic patch is found on the opposite site in loops 5 and 6. Mutated amino acids, e.g., in [V10K] and [T20K] kalata B1 were modeled using the PyMol mutation wizard and have been shown in red color in the enlarged window. All Figure representations were prepared using PyMol.

Native kalata B1, a cyclotide isolated from *Oldenlandia* *affinis* DC. (*Rubiaceae*), was purified from aerial plant parts. Several linear precursors of single point-mutated lysine or alanine analogues of kalata B1 were assembled using solid-phase peptide synthesis. After cleavage from the resin and purification with reversed-phase high performance liquid chromatography (RP-HPLC), the mutant peptides were cyclized and oxidized. The yields of the kalata B1 mutants [T8K], [V10K], [V10A], [G18K], [T20K] and [N29K] after oxidative folding were calculated to 15%, 16%, 21%, 22%, 40% and 29%, respectively, which are of similar magnitude to synthetic cyclotide yields described previously [37,38]. The yield of cyclization was calculated by comparing the peak area of the correctly folded peptide to the total peak area of the analytical RP-HPLC chromatogram. The HPLC profile of the representative mutant [T20K] kalata B1 is illustrated in Figure S1. NMR α-H chemical shifts of all mutants were determined using two-dimensional NMR spectra and showed no significant differences from kalata B1, indicating that the mutant cyclotides had similar three-dimensional folds to wild-type kalata B1. The amino acid sequence and structure, including the disulfide connectivity, of kalata B1 is shown in Figure 1 and the positions of the mutations are indicated. Exemplarily, a chemical shift comparison of kalata B1, [V10K] kalata B1 and [T20K] kalata B1 is shown in Figure 1B. Furthermore, space-filling structural models of the [V10K] and [T20K] mutants and kalata B1 indicated that the replaced residues are located on opposite sides of the peptide surface and that the valine to lysine or threonine to lysine mutation, respectively, slightly affected the area and hydrophobic properties of the surface of the cyclotides (Figure 1C).

### Structure-activity relationships of cyclotides on lymphocyte and isolated T-cell proliferation

**Figure 2:**
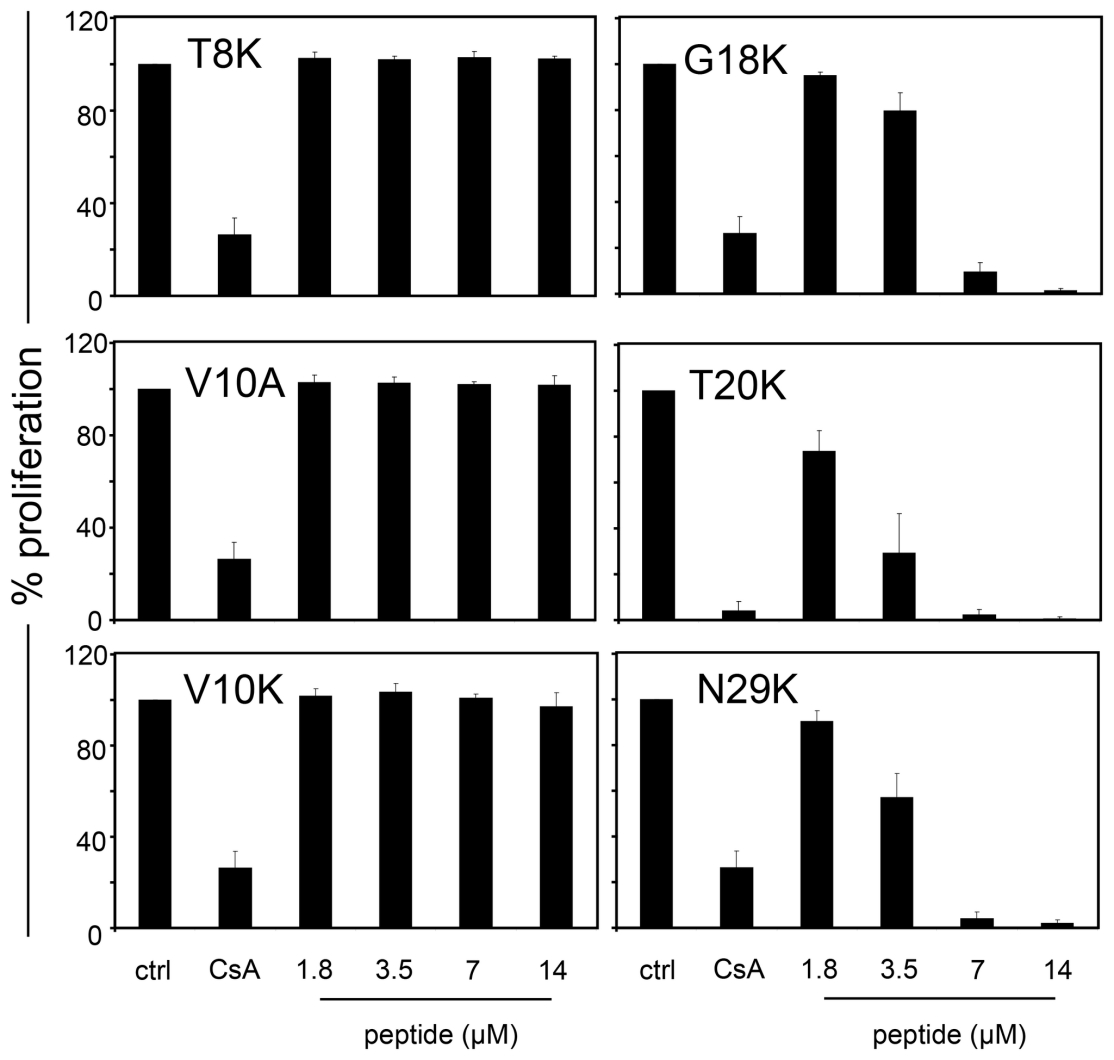
Effects of cyclotide mutants on cell proliferation of primary activated human lymphocytes. The influence of medium (ctrl), CsA (0.8 µM) or different concentrations of the kalata B1 cyclotide mutants [T8K], [V10A], [V10K], [G18K], [T20K] and [N29K] (1.8-14 μM) on proliferation of activated primary lymphocytes was measured by cell division analysis using CFSE-based flow cytometry on day three post stimulation. Data are presented as mean ± SD of four (three for [T20K] kalata B1) independent donors and experiments. Corresponding IC_50_ values have been presented in Table 1.

We recently reported [11] that kalata B1 has anti-proliferative activity on PBMCs. To determine the cyclotide motif responsible for the anti-proliferative capacity, several kalata B1 mutants were analyzed by stimulation of CFSE-labeled lymphocytes, or purified T-cells in the presence of control medium, camptothecin (30 µg/mL), CsA (0.8 µM) or various concentrations of cyclotides (1.8-14 µM). The data indicated that the kalata B1 mutants [G18K], [N29K] and [T20K] exhibited a dose-dependent anti-proliferative function on lymphocytes (Figure 2), as well as on purified T-cells (Figure S2) with corresponding IC_50_ values ranging from 1.9 to 4.4 µM (Table 1); no signs of cytotoxicity were observed in the analyzed concentration range up to 14 µM, which is in agreement with a previous study [11]. In contrast, the mutants [T8K] kalata B1, [V10A] kalata B1 and [V10K] kalata B1 did not suppress the proliferation of T-cells and one representative cyclotide ([V10K]) was thus further used as an ‘inactive’ control in the mechanistic signaling studies.

**Table 1:** Potency of kalata B1 and mutants to inhibit lymphocyte and purified T-cell proliferation.

**Peptide**	**IC_50_ (µM)** ± **SD**

*Lymphocytes (PBMCs)*	
**native kalata B1**	2.9 ± 1.3^a^
[T8K]	not active (n.a.)^b^
[V10A]	n.a.^b^
[V10K]	n.a.^b^
[G18K]	4.4 ± 0.5^b^
[T20K]	1.9 ± 0.6^c^
[N29K]	3.2 ± 0.6^b^
*Purified T-cells*	
**native kalata B1**	2.4 ± 0.5^d^
[G18K]	3.2 ± 1.8^c^
[T20K]	2.7 ± 0.6^d^
[N29K]	2.1 ± 0.9^c^

data have been normalized and analyzed with non-linear regression (fixed slope) using Graph Pad Prism, a: n=7, b: n=4, c: n=3, d: n=2; n.a. not active; peptides other than kalata B1 are amino acid mutants of the native cyclotide and position of mutations are indicated by numbers (see Figure 1)

Recent studies identified distinct sub-regions of the kalata B1 molecule that are associated with its mode-of-action, namely (i) a hydrophilic face centered around the residue Glu-7 and adjacent residues, (ii) a surface-exposed hydrophobic patch formed by residues Leu-2, Pro-3, Val-4, Val-10, Trp-23, Pro-24 and Val-25 and (iii) an amendable face formed by residues Gly-1, Gly-18, Thr-20, Ser-22, Thr-27 and Asn-29 [37,38]. Our results support this clustering phenomenon and suggest that it may be a general mechanism for other biological activities of cyclotides. The hydrophilic face has been described previously as the ‘bioactive face’, since disruption of its physico-chemical properties by mutation of amino acids to alanine or lysine leads to a loss-of-function [37]. Accordingly, the kalata B1 mutants [G8K], [V10K] and [V10A] (Figure 1) lost their immunosuppressive activity (Table 1 Figure 2). On the other hand, mutations of residues Gly-18, Thr-20 and Asn-29 to lysine, which are located on the amendable face of the molecule (Figure 1), did not negatively affect anti-proliferative capacity on lymphocytes and isolated T-cells, relative to native kalata B1. This observation is in agreement with previous studies on the anthelmintic activity of cyclotides [37,39].

It is evident that single amino acid replacements within the ‘active’ cyclotide face and in particular of residues Gly-8 and Val-10 have detrimental effects on immunosuppressant activity, suggesting compound specificity. Additionally, analysis of a cyclotide all-D-enantiomer in comparison with the native L-form suggests a direct stereospecific target interaction. Specifically, all-D-kalata B2 had a significantly reduced anti-proliferative effect on PBMCs and purified T-cells at concentrations of 1.8 and 3.5 µM, respectively, in comparison to native kalata B2 [11] (Figure S3). Moreover, proliferation analysis of activated lymphocytes *versus* purified T-cells suggested that the cyclotide-mediated immunosuppression was of direct origin, and no anti-proliferative activity changes were observed between bulk lymphocytes and purified T-cells (Table 1). Knowing that the immunosuppressive activity of cyclotides was compound-specific, stereospecific and directly related to T-cell biology, the kalata B1 mutants [T20K] (‘active’) and [V10K] (‘inactive’) were further analyzed regarding signaling pathways of T-cell proliferation in comparison to the well-characterized immunosuppressant drug CsA.

### Influence of cyclotides on interleukin 2-receptor expression

**Figure 3:**
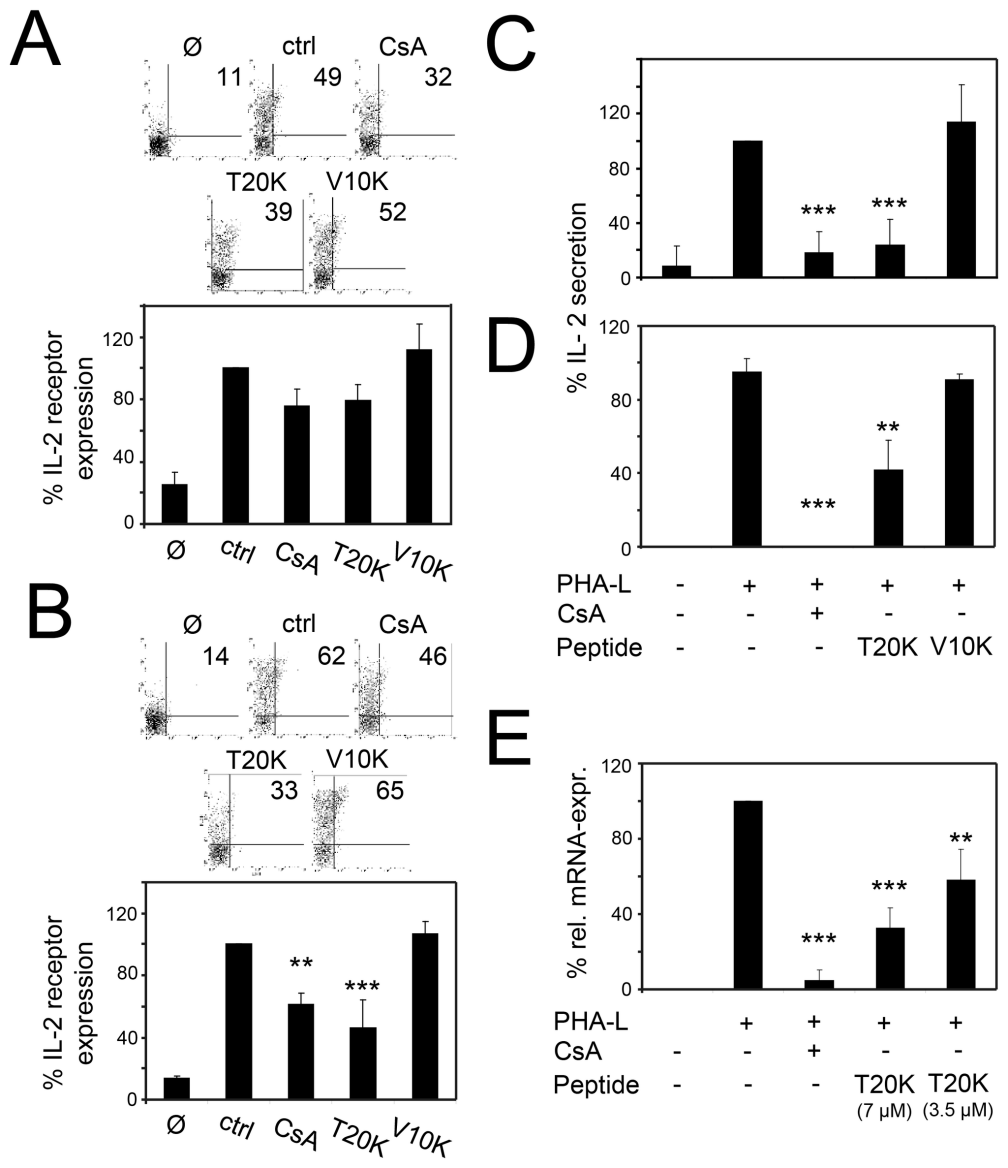
Effects of cyclotide mutants on IL-2 biology of primary activated human lymphocytes and purified T-cells. For IL-2 receptor expression studies, lymphocytes were treated with CsA or cyclotides (4 µM each) and were cultivated in the presence of media (Ø) or activation stimuli alone (ctrl). At 24 h (**A**) or 36 h (**B**) after cultivation, cells were surface-stained with anti-human CD25 mAbs and were analyzed by flow cytometry. For IL-2 secretion analysis, lymphocytes (**C**) or purified T-cells (**D**) were restimulated with PMA (50 ng/mL) and ionomycin (500 ng/mL) for 6 h after 24 h of cultivation. Afterwards, the amount of IL-2 was individually measured in the supernatant by using ELISA-based techniques. The *il-2* gene expression of lymphocytes was analyzed by quantitative RT-PCR (**E**). The data were normalized to the cycle threshold value of the internal housekeeping gene *18s rRNA* and the relative mRNA level in the untreated stimulated group was used as calibrator. Data are expressed as mean ± SD of independent donors and experiments as indicated. For IL-2 receptor analysis representative data were additional depicted as dot plots. The asterisks represent significant differences (*P <0.05, **P <0.01, ***P <0.001) of treated cells in comparison to ctrl (PHA-L stimulated cells alone).

Amongst other pathways, T-cell proliferation is initiated by ligation of the T-cell receptor to antigens that trigger a complex T-cell receptor signaling pathway. During this process, T-cells express the autocrine growth factor interleukin 2 (IL-2), which promotes interaction with its surface receptor that is up-regulated in activated T-cells [40]. Therefore the influence of cyclotides on the expression of the IL-2 receptor was analyzed using [T20K] kalata B1 and [V10K] kalata B1. Treatment of lymphocytes with CsA or [T20K] kalata B1 led to a reduction of the IL-2 surface receptor CD25 expression (76% ± 11 or 79% ± 10, respectively) after 24 h as compared to untreated cells, i.e., stimulated lymphocytes (ctrl, 100%) (Figure 3A). This observation is even more significant for 36 h of treatment, i.e., the CD25 expression was further reduced to 62% ± 7.3(CsA) and 46% ± 18 ([T20K] kalata B1), respectively (Figure 3B). Thus, the IL-2 receptor expression analysis of activated T-cells demonstrated a reduction of the cell surface expression of the receptor in the presence of the immunosuppressive cyclotide [T20K] kalata B1. The inhibition over time was comparable to that of CsA and these results confirm the potential of CsA to influence the early activation state of lymphocytes, due to partial suppression of the IL-2 receptor [41,42]. The receptor surface level of the cells that were treated with the inactive cyclotide mutant [V10K] was unaffected.

Since calcium is an important messenger and involved in T-cell receptor signaling, we tested whether cyclotides had an effect on Ca^2+^-release. Purified T-cells were treated with CsA, [T20K] kalata B1 or [V10K] kalata B1 (4 µM each) and neither cyclotide induced any direct changes in Ca^2+^-flux. Similarly, any inhibitory effects on the release of Ca^2+^ by cyclotide pretreatment were measured; T-cells were incubated with CsA, [T20K] kalata B1 or [V10K] kalata B1 overnight and Ca^2+^-release was triggered by adding phorbol myristate acetate (PMA) and ionomycin to the pretreated cells during flow cytometric measurement. Neither cyclotides nor CsA induced any inhibition or a delay in Ca^2+^-signaling (Figure S4) and this observation provided evidence for a downstream mechanism-of-action of immunosuppressive cyclotides.

### Influence of cyclotides on IL-2 release and gene expression

Besides IL-2 receptor expression, T-cell proliferation is regulated by endogenous release of IL-2. It is accepted that CsA inhibits the production of IL-2 [43] and because IL-2 is a pivotal lymphokine during immune responses, inhibition of its production may explain the immunosuppressive effects of [T20K] kalata B1. Therefore the capacity of cyclotides to influence the direct release of IL-2 from lymphocytes (Figure 3C) or purified T-cells (Figure 3D) was determined. Cells were treated with [T20K] kalata B1, [V10K] kalata B1 or CsA and were activated using mitogen stimulation for 24 h followed by re-stimulation with PMA and ionomycin. The IL-2 release of T-lymphocytes was significantly reduced (*P* <0.01) by treatment with CsA (18% ± 16) and [T20K] kalata B1 (24% ± 19) compared to the control cells (Figure 3C), whereas [V10K] kalata B1 had no effect on the release of IL-2. Moreover, the supernatants of stimulated purified T-cells were analyzed for their IL-2 release capacity and it was evident that [T20K] kalata B1 significantly decreased the release of IL-2 relative to [V10K] kalata B1 (Figure 3D). To determine whether cyclotides have an impact on the transcriptional level, the *il-2* gene expression was analyzed by quantitative real-time PCR (Figure 3E), relative to the *18s rRNA* control transcript. Both CsA and [T20K] kalata B1 clearly decreased the level of *il-2* mRNA compared to the control.

**Figure 4:**
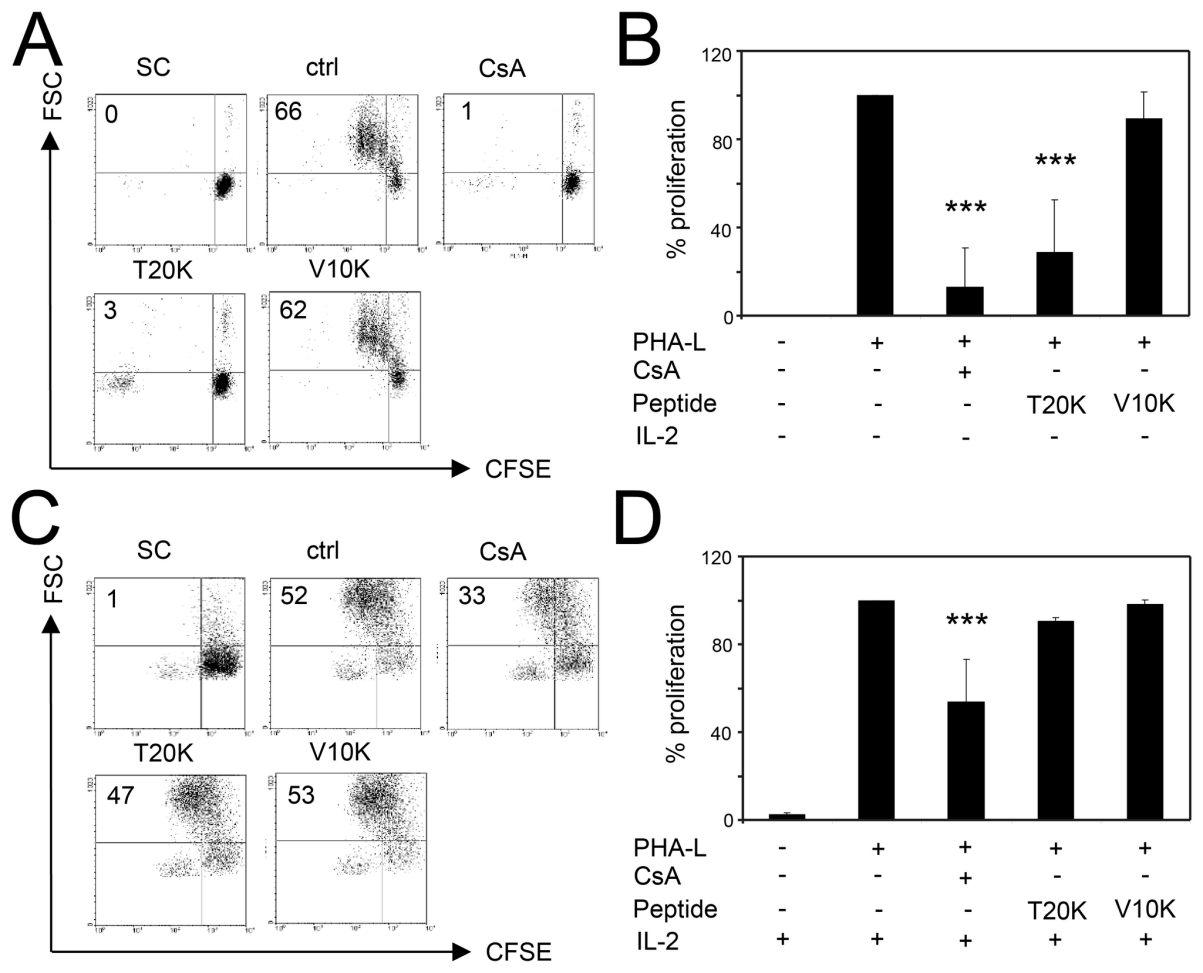
Effects of exogenous IL-2 on proliferation capacity of cyclotide-treated lymphocytes. CFSE-labeled primary human lymphocytes were pretreated with CsA or cyclotides (4 µM each) and were activated using PHA-L (10 µg/mL), followed by washing off the cyclotides and activation stimuli. Furthermore, the cells were cultured without (**A** and **B**) or in the presence of exogenous recombinant human IL-2 (**C** and **D**). The proliferation capacity of the cells was analyzed at day three by flow cytometry. Representative data are presented in dot plots (**A** and **B**) and summary of three independent experiments were presented as mean ± SD (**B** and **D**). The values in dot plots represents the amount of proliferated lymphocytes on which the quantification to control is based. The asterisks represent significant differences of treated cells in comparison to ctrl (PHA-L stimulated cells alone) (*P <0.05, **P <0.01, ***P <0.001).

The data demonstrated that treatment with [T20K] kalata B1 reduced IL-2 release of activated lymphocytes and purified T-cells. The notion that cyclotide-induced immunosuppression is mediated by an IL-2-dependent inhibition of proliferation is further supported by the observation of the reduced transcriptional IL-2 level. To determine the validity of the significant IL-2 reduction after cyclotide treatment, the influence of exogenous addition of IL-2 post treatment was analyzed. If inhibition of IL-2 production is the exclusive mode-of-action of CsA and cyclotides, one would expect that this effect is reversible by exogenous addition of IL-2 to the cell culture medium. For this purpose, the cells were preincubated with cyclotides or CsA before activation. After the activation period, the cells were washed to remove any excess of cyclotides or CsA, respectively (Figure 4A and B). In parallel, the cells were treated and cultured simultaneously with the addition of exogenous IL-2 (Figure 4C and D). Pretreatment of cells with CsA and [T20K] kalata B1 led, as expected, to an anti-proliferative effect (13% ± 18 and 29% ± 24, respectively) compared to control cells, whereas treatment with [V10K] kalata B1 had no effect on proliferation (Figure 4B and D). By adding exogenous IL-2 it was possible to reverse the anti-proliferative effect of CsA partially (54% ± 19) and of [T20K] kalata B1 completely (91% ± 1.4) (Figure 4C and D). Accordingly, the supplementation of IL-2 to the [V10K] kalata B1-treated lymphocytes did not have any effect.

The addition of IL-2 to cultures still containing [T20K] kalata B1 throughout the whole cultivation period was ineffective at restoring normal proliferation. Our findings demonstrated that only cells preincubated with [T20K] kalata B1, which had been thoroughly washed to remove any peptides or stimuli, were sensitive to the addition of exogenous IL-2 by restoring their normal proliferation rate.

### Influence of cyclotides on the effector function of activated T-lymphocytes

**Figure 5:**
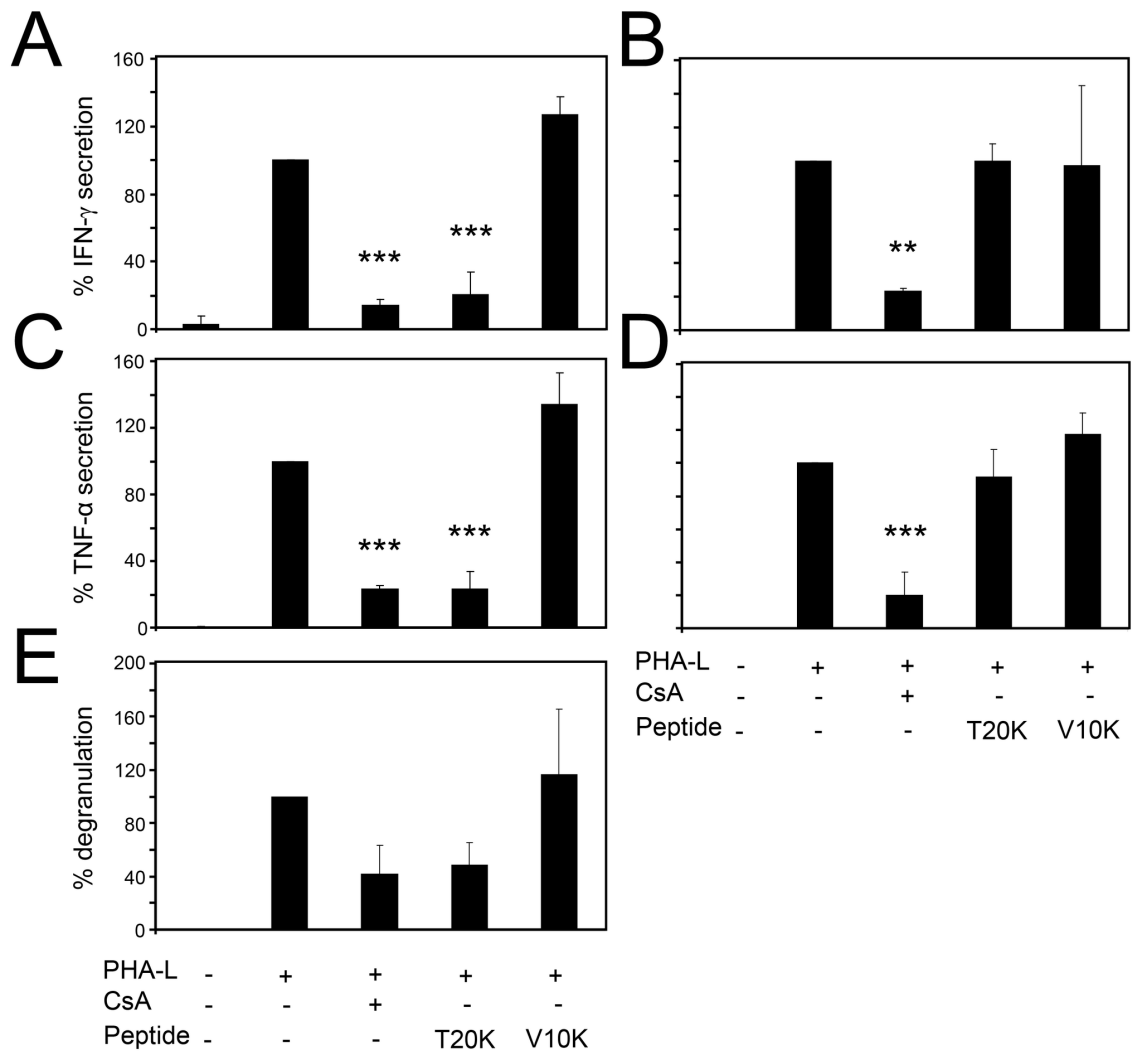
Influence of cyclotide mutants on effector function of primary activated human lymphocytes. Purified lymphocytes were preincubated with CsA or cyclotides (4 µM each), then stimulated with PHA-L (10 µg/mL) and exposed to a re-stimulation impulse with PMA and ionomycin for 6 h before analysis of effector functions, expressed as amounts of IFN-γ (24 h: **A**; 36 h: **B**), TNF-α (24 h: **C**; 36 h: **D**) or degranulation capacity (**E**). The amount of IFN-γ and TNF-α was measured in the supernatant of cultured cells using an ELISA-based method for early time points, or intracellular cytokine detection flow cytometric analysis for late time points. The degranulation capacity was detected by using classical CD107a assay. Data of three independent experiments were presented as mean ± SD. The asterisks represent significant differences of treated cells in comparison to ctrl or PHA-L stimulated cells alone (*P <0.05, **P <0.01, ***P <0.001).

The results from the quantitative analysis presented above demonstrated that treatment of activated lymphocytes with CsA or [T20K] kalata B1 influences expression of the IL-2 surface receptor and IL-2 secretion of lymphocytes and purified T-cells. Consequently it was of interest to determine whether cyclotides solely inhibit proliferation or affect the effector function of T-lymphocytes, which would directly relate to changes in interferon-gamma (IFN-γ) and tumor necrosis factor-alpha (TNF-α) levels. Therefore, the production of both mediators was analyzed following treatment of activated T-lymphocytes with cyclotides or CsA (Figure 5). The IFN-γ concentration of the CsA and [T20K]-treated cells at an early time point (i.e., 24 h post stimulation) was significantly reduced to 14% ± 3.4 and 21% ± 13, respectively, as compared to the control. The production of IFN-γ was not affected by [V10K] kalata B1 (Figure 5A). Accordingly, treatment with CsA (23% ± 1.8) or [T20K] kalata B1 (23% ± 11) resulted in a significantly reduced TNF-α expression (Figure 5C). To determine whether the effector function of T-lymphocytes remained compromised, IFN-γ and TNF-α release was measured at a later time-point, i.e., 36 h post stimulation (Figure 5B and D). These experiments indicated that treatment with CsA resulted in a prolonged reduction of IFN-γ (23% ± 1.9), whereas the cyclotide [T20K]-treated cells had the ability to recover over time, i.e., the cyclotide treatment had no effect on the IFN-γ level after 36 h (Figure 5B). Similarly, TNF-α production was reduced after treatment with CsA (20% ± 14) whereas the cyclotide-treated cells ([T20K] kalata B1 and [V10K] kalata B1) did not affect the TNF-α level at the late time point after activation (Figure 5D). The capacity to diminish the release of these mediators from activated cells by [T20K] kalata B1 is comparable to that of CsA. Treatment with [T20K] kalata B1 led to an initial reduction of the effector function, as indicated by the reduced IFN-γ and TNF-α production, but the levels of both cytokines stabilized over time and returned to their normal levels. This further indicated that the cyclotide [T20K] kalata B1 and CsA have a different mechanism-of-action.

After determining the influence of cyclotide treatment on the effector function of T-lymphocytes, it was of interest to determine cyclotide-induced changes on the degranulation activity. Activation of T-lymphocytes leads to a release of cytolytic granules that contain lysosomal-associated membrane protein 1 (CD107; LAMP-1). During degranulation, the granule vesicle membranes fuse with the membranes of activated T-lymphocytes and therefore LAMP-1 can be used as a marker protein for the cytotoxic activity of these cells. After 36 h, 42% ± 21 of the CsA and 49% ± 17 of the [T20K] kalata B1-treated cells contained the degranulation marker LAMP-1 as compared to the control (Figure 5E), which demonstrated that either treatment reduced degranulation capacity, whereby the cyclotide [V10K] kalata B1 had no influence on this functional activity of stimulated T-lymphocytes. This indicated that cyclotide-treated cells restored their polyfunctionality in contrast to CsA-treated controls. The TNF-α gene is one of the earliest to be transcribed in lymphocytes after activation, followed by the production of both IL-2 and IFN-γ. Such process defines a significant part of the functional activity of a T-cell and the ability to produce multiple cytokines has been associated with beneficial immune responses [44]. Polyfunctionality additionally has clinical significance because reduction of the cytokines IL-2, TNF-α and IFN-γ by immunosuppressive treatment is associated with an increased risk of infection [45].

## Conclusion

Cyclotides are an abundant and diverse natural combinatorial library of cyclic cystine knot peptides that exhibit great stability. We characterized the immunosuppressive structure-activity relationships of cyclotides using assays to determine their anti-proliferative effects on cells of the human immune system (lymphocytes). The active [T20K] and inactive [V10K] kalata B1 mutants were used for detailed mechanistic immunological studies and demonstrated that cyclotides suppress T-cell polyfunctionality and arrest the proliferation of immune-competent cells through inhibiting IL-2 biology at more than one site. The effect was stereospecific indicating a direct cyclotide-target interaction. As a consequence these results open new avenues to utilize synthetically-optimized cyclotides for *in vivo* applications in immune-related disorders and as immunosuppressant peptide drugs. The oral activity and presumed oral bioavailability of cyclotides [32], the availability of recombinant and synthetic production techniques [46,47] as well as the plasticity of the cystine-knot framework, which is amenable to a wide range of amino acid substitutions, provides a promising basis for future studies in animal model systems related to malfunctioning of immune cells in general and in particular the over-reactivity of T-cells.

## Materials and Methods

### Extraction of cyclotides from plants

Native kalata B1 and kalata B2 were extracted and purified from aerial parts of the Rubiaceae plant *Oldenlandia* *affinis* DC. as described earlier [11].

### Peptide synthesis and analysis

The linear precursors of the kalata B1 lysine mutants [T8K], [V10K], [G18K], [T20K] and [N29K] and the alanine mutant [V10A] containing an N-terminal Cys residue and a C-terminal thioester linker were synthesized using manual solid-phase peptide synthesis with an *in situ* neutralization/HBTU protocol [48] for Boc chemistry on a 0.5 mM scale [37,38]. The all-D-enantiomer of kalata B2 was synthesized using D-amino acid building blocks according to Henriques et al. [49]. Following resin cleavage with hydrogen fluoride, backbone cyclization of the linear peptides was achieved through native chemical ligation to form a peptide bond between two ends [50]. Cyclization and oxidative folding were performed in a one-step reaction by dissolving the peptides at 0.5 mg/mL in folding buffer containing 50% isopropyl alcohol (v/v) in 0.1 M ammonium bicarbonate (pH 8.2) with the addition of 2 mM reduced glutathione and 0.4 mm oxidized glutathione. The mixture was stirred for 48 h at 23°C and the peptide solutions were diluted with 0.1% trifluoroacetic acid and purified by preparative RP-HPLC. The correctly folded mutants were identified and characterized by analytical RP-HPLC and mass spectrometry. Correct folding of the peptides was confirmed by NMR spectrometry and samples contained 1–2 mM peptide in 90% H_2_O/10% D_2_O (v/v). One- and two-dimensional NMR spectra of mutants were recorded on Bruker Avance 500 MHz or 600 MHz spectrometers at 298 K as described previously [51].

### Ethics statement

All experiments conducted on human material were approved by the Ethics committee of the University of Freiburg (235/11; 22.06.11).

### Preparation and cultivation of human peripheral lymphocytes or purified T-cells

Human peripheral lymphocytes were isolated from the blood of healthy adult donors obtained from the Blood Transfusion Centre (University Medical Center, Freiburg, Germany). Venous blood was centrifuged on a LymphoPrep^TM^ gradient (density: 1.077 g/cm^3^, 20 min, 500 x g, 20°C; Progen, Heidelberg, Germany). Purified T-cells were obtained by CD3^+^ positive selection using magnetic cell separation method according to manufacturer’s instructions (StemCell Technologies, Grenoble, France). Cells were washed twice with medium and cell viability as well as concentration was determined using the trypan blue exclusion test. Cells were cultured in RPMI 1640 medium supplemented with 10% heat-inactivated fetal calf serum (PAA, Pasching, Austria), 2 mM L-glutamine, 100 U/mL penicillin and 100 U/mL streptomycin (all from Life Technologies, Paisley, UK). The cells were cultured at 37°C in a humidified incubator with a 5% CO_2_/ 95% air atmosphere.

### Activation and treatment of lymphocytes and T-cells

Lymphocytes were either stimulated with anti-human CD3 (clone OKT3) and anti-human CD28 (clone 28.2) mAbs (each 100 ng/mL; both from eBioscience, Frankfurt, Germany) or phytohemagglutinin-L (PHA-L, 10 µg/mL; Roche Diagnostics, Basel, Switzerland) as indicated in the presence of medium, CsA (0.8 or 4 µM, respectively; Sandimmun^®^ 50 mg/mL, Novartis Pharma, Basel, Switzerland), camptothecin (CPT; 30 µg/mL: Tocris, Bristol, UK) and 0.5% Triton-X 100, or cyclotides. After cultivation, the cells were assessed in biological tests as described. Alternatively for IL-2 supplementation assays, lymphocytes or purified T-cells were equilibrated for 2 h at 37°C. Afterwards, cells (4 x 10^5^) were preincubated for 2 h with CsA or cyclotides, transferred to a new plate and stimulated with

10 µg/milliliter PHA-L for 1 h. This was followed by washing off the substances and stimuli with PBS and resuspending the cells in medium for further assays as described.

### Determination of cell proliferation and cell division

For cell proliferation and cell division tracking analysis, cells were harvested and washed twice in cold PBS and resuspended in PBS at a concentration of 5 x 10^6^ cells/mL. Cells were incubated for 10 min at 37°C with carboxyfluorescein diacetate succinimidyl ester (CFSE; 5 µM: Sigma-Aldrich, St. Louis, MO). The staining reaction was stopped by washing twice with complete medium and the cell division progress was analyzed by flow cytometric analysis using a FACSCalibur instrument (BD Biosciences, Becton Dickinson, Franklin Lakes, NJ).

### IL-2 surface receptor analysis

Cultured cells were washed with PBS and stained with PE-labeled anti-human CD25 mAbs (eBioscience) for 15 min at 4°C. Afterwards, the cells were washed twice with PBS and resuspended and transferred into FACS vials and expression of the IL-2 surface receptor α-chain CD25 was measured by FACS analysis using a FACSCalibur instrument.

### Determination of cytokines and degranulation analysis

Cells were treated for 24 or 36 h, respectively and were restimulated with PMA (50 ng/mL) and ionomycin (500 ng/mL) for additional 6 h. Supernatants were harvested by centrifugation and were stored at -20°C. The amount of cytokines was measured and quantified using the FlowCytomix^TM^ technique according to manufacturer’s instructions (eBioscience) or alternatively intracellular cytokine production was determined. For this purpose the cells were fixed and permeabilized using 4% paraformaldehyde (Sigma-Aldrich) and Perm/Wash solution (Becton Dickinson), followed by staining with PE-conjugated anti-human IFN-γ mAb or anti-human TNF-α mAb (both from eBioscience), respectively. For CD107a degranulation assay, cells were restimulated with PMA (10 or 50 ng/mL, respectively) und ionomycin (100 or 500 ng/mL, respectively) and to each well containing 200 µL of cell suspension, 5 µL (~0.25 µg) PE-conjugated anti-CD107a mAb (eBioscience) were added. After incubation at 37°C for 1 h, 2 µL of 1/10 diluted GolgiStop (Becton Dickinson) was added per well and the plates were incubated for another 2.5 h. Samples were analyzed with a BD FACSCalibur flow cytometer using BD CellQuest Pro Software.

### Total RNA extraction, reverse transcription and real-time PCR

Total RNA was extracted from controls or treated cells (4 x 10^6^) and frozen at -80°C until further use. RNA purification was performed according to the manufacturer’s instructions for the RNeasy mini and RNAse-Free DNAse digestion kits (Qiagen, Hamburg, Germany). The quantity and purity of extracted RNA was measured by nanodrop spectrophotometry (Peqlab, Erlangen, Gemany) and purified RNA was reverse transcribed using the RT^2^ First Strand Kit (Qiagen). RT-PCR reactions were carried out on a MyiQ kit (BioRad, Munich, Germany) in a final volume of 25 µL using RT² qPCR Primer Assay (for *il-2*) and SYBR^®^ Green qPCR master mix (both from Qiagen). Each determination was performed in duplicate and the housekeeping gene *18s rRNA* was used as an internal control. The real-time thermal cycler program comprised an initial denaturation step at 95^°^C for 10 min followed by a two-step program with 40 cycles (95°C, 15 sec; and 60°C, 60 sec). The relative gene expression of *il-2* has been determined by comparative cycle threshold analysis and the data were normalized to the internal housekeeping gene *18s rRNA* and the relative mRNA level in the untreated group (untreated PHA-L-activated).

### Ca^2+^-release assay

Human primary T-cells (1 x 10^6^/mL) were loaded with 1 µM Fura-2 and 0.02% Puronic F-127 for 30 min at 37°C. Cells were centrifuged for 5 min at 1200 rpm and resuspended in RPMI 1640 media supplemented with 10% fetal calf serum, penicillin (100 U/mL) and streptomycin (100 U/mL). 100 µL of cell suspension were transferred into a black 96-well plate with a clear flat-bottom. Briefly before analysis the fluorometer Synergy H4 (BioTek, Winooski, VT) was equilibrated to 37°C. The fluorescence time course was measured with: extinction at 340 and 380 nm, respectively, and emission at 510 nm in 30 sec intervals, while continuously shaking. Ca^2+^-influx was initiated by adding compounds (CsA, [T20K] kalata B1 and [V10K] kalata B1; 4 µM each) to the cells. To receive maximum Ca^2+^-release, cells were stimulated with PMA (50 ng/mL) and ionomycin (500 ng/mL). For lowest Ca^2+^-levels, cells remained untreated. To detect an inhibitory effect induced by cyclotides in PMA and ionomycin stimulated cells, T-cells (4 x 10^6^/mL) were incubated with CsA, [T20K], [V10K] (4 µM each) or left untreated over night at 37°C in a 24-well flat-bottom plate. Cells were stained for 15 min with Fluo-4 (2 µM) and Fura Red (4 µM), centrifuged at 1000 rpm for 5 min and resuspended in medium. Ca^2+^-release was measured by time-dependent flow cytometry using BD FACSCalibur as described previously [52]. Fluo-4 and Fura Red signals were detected using flow cytometric analysis. After equilibrating basal calcium levels for 60 sec, PMA (50 ng/mL) and ionomycin (500 ng/mL) were added to the preincubated T-cells. Fluorescence ratio was calculated using WEASEL 2.3 software.

### Data- and statistical analysis

For statistical analysis, data were processed with Microsoft Excel, GraphPad Prism and SPSS software. Values are presented as mean ± SD for the indicated number of independent experiments. Statistical significance was determined by one-way ANOVA followed by Dunnett’s post hoc pairwise comparisons, unless otherwise stated. Statistical differences in cell proliferation between the cyclotide enantiomers kalata B2 and D-kalata B2 were analyzed by inspection of homogeneity of variance between groups as assessed by Levene’s Test for Equality of Variances, followed by an independent t-test. *P* values <0.05 are considered as statistically significant.

## Supporting Information

Figure S1 Folding/cyclization and purification of [T20K] kalata B1. (**A**) HPLC profile of [T20K] kalata B1 after a one-pot oxidation/cyclization procedure. The late eluting peak (indicated with an asterisk) was confirmed to be the correctly folded [T20K] by NMR spectroscopy (Figure 1). (**B**) The purity of [T20K] kalata B1 was evaluated by analytical RP-HPLC. The sharp and symmetrical peak suggests that the purity of T20K is >95%.
(TIF)

Figure S2 Effects of cyclotide mutants on cell proliferation of activated primary purified human T-cells. The proliferation capacity of CFSE-labeled primary purified T-cells were analyzed at day 3 in the presence of medium (ctrl), camptothecin (CPT, 30 µM), cyclosporin A (CsA, 0.8 µM) or different concentrations of the kalata B1 cyclotide mutants (1.8-14 µM). Data are presented as mean ± SD of three (two for [T20K] kalata B1) independent donors and experiments. The corresponding IC_50_ values have been presented in Table 1.
(TIF)

Figure S3 Effects of a cyclotide all-D-enantiomer on cell proliferation of primary activated human lymphocytes and purified T-cells. The influence of medium (ctrl), or kalata B2 and its all-D-enantiomer (D-kalata B2) at various concentrations (1.8-7 µM) on proliferation of activated primary lymphocytes (**A**) and purified T-cells (**B**) was measured by cell division analysis using CFSE-based flow cytometry on day three post stimulation. Data are presented as mean ± SD of three independent donors and experiments (*P <0.05; n.s. not significant).
(TIF)

Figure S4 Ca^2+^-release in human T-cells. (**A**) The fluorescence time course of Fura-2 loaded human primary T-cells was measured by extinction at 340 and 380 nm, respectively and emission at 510 nm in 30 sec intervals, while continuously shaking. Ca^2+^-influx was initiated by adding compounds to the cells (illustrated by the arrow). To receive maximum Ca^2+^-release, cells were triggered with PMA (50 ng/mL) and ionomycin (500 ng/mL) (reversed triangles). For lowest Ca^2+^-levels, cells remained untreated (diamond-shapes, dotted line). Stimulation with [T20K] kalata B1 (open squares), [V10K] kalata B1 (open triangles) or cyclosporin A (open circles) (4 µM each) did not induce any change in Ca^2+^-signaling. (**B**) Ca^2+^-flux was additionally analyzed by FACS using Fluo-4 and Fura Red labeled T-cells, which were pretreated with cyclosporin A or cyclotides for 16 h. None of the compounds [T20K] kalata B1, [V10K] kalata B1 or cyclosporin A induced an inhibition of Ca^2+^-release after addition of PMA and ionomycin (indicated by the arrow).
(TIF)
