# Supplementary figures and images for: Cyclotides Suppress Human T-Lymphocyte Proliferation by an Interleukin 2-Dependent Mechanism
Source: PLoS One. 2013 Jun 26;8(6):e68016. doi: 10.1371/journal.pone.0068016 (PMC3694003; doi:10.1371/journal.pone.0068016)

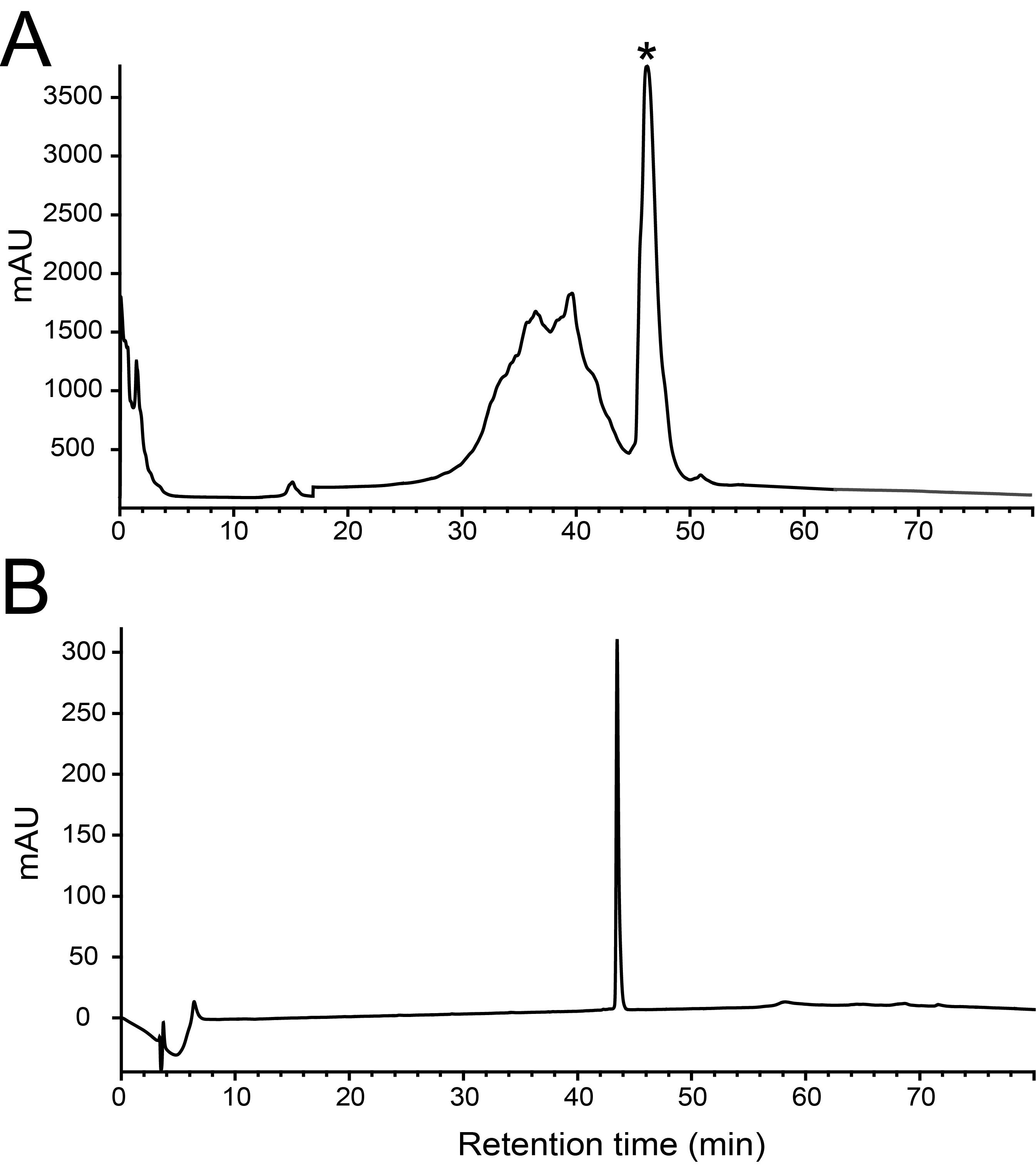

Supplement: Figure S1 — (A) HPLC profile of [T20K] kalata B1 after a one-pot oxidation/cyclization procedure. The late eluting peak (indicated with an asterisk) was confirmed to be the correctly folded [T20K] by NMR spectroscopy (Figure 1). (B) The purity of [T20K] kalata B1 was evaluated by analytical RP-HPLC. The sharp and symmetrical peak suggests that the purity of T20K is >95%. (TIF) [file pone.0068016.s001.tif]

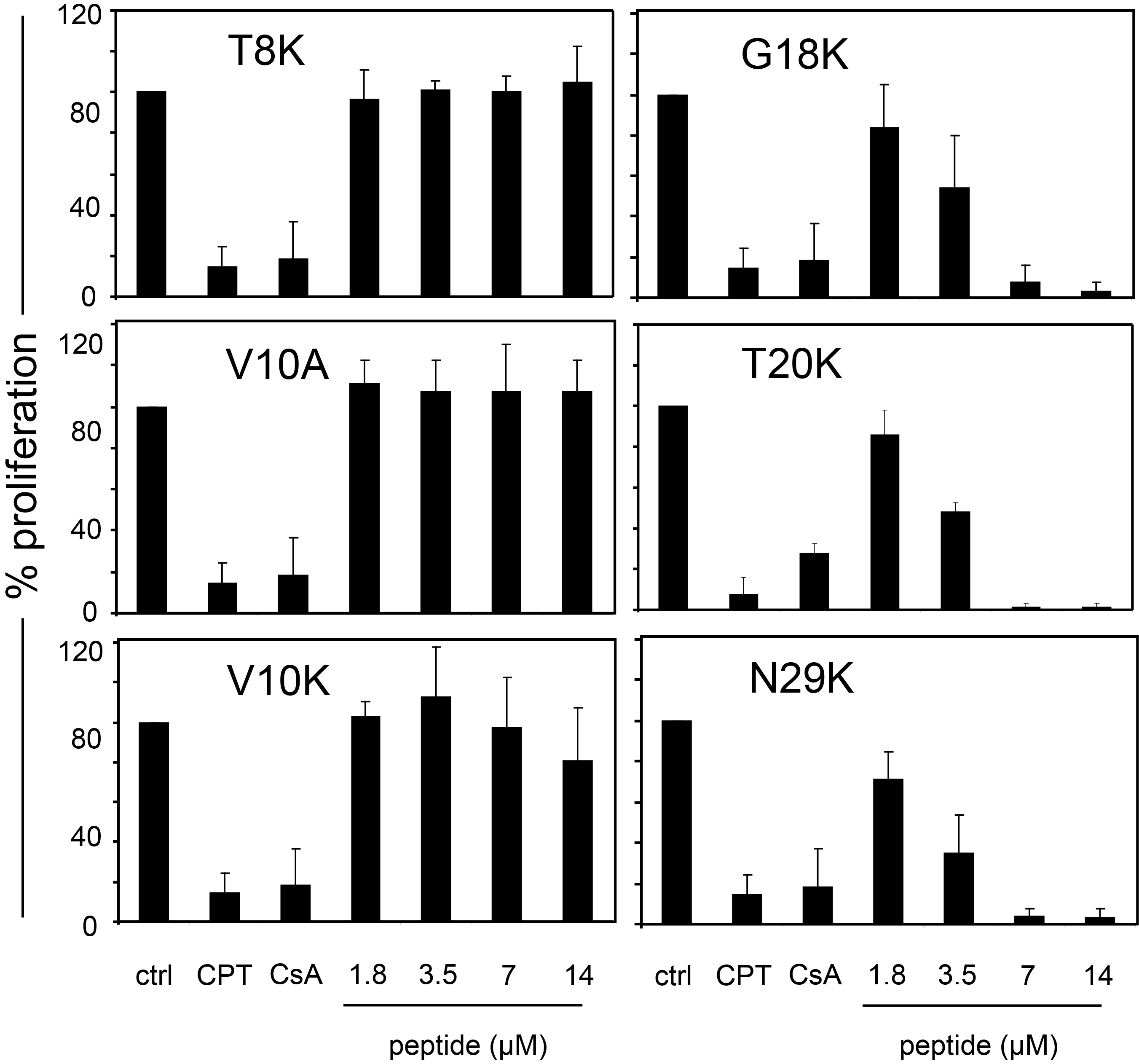

Supplement: Figure S2 — The proliferation capacity of CFSE-labeled primary purified T-cells were analyzed at day 3 in the presence of medium (ctrl), camptothecin (CPT, 30 µM), cyclosporin A (CsA, 0.8 µM) or different concentrations of the kalata B1 cyclotide mutants (1.8-14 µM). Data are presented as mean ± SD of three (two for [T20K] kalata B1) independent donors and experiments. The corresponding IC50 values have been presented in Table 1. (TIF) [file pone.0068016.s002.tif]

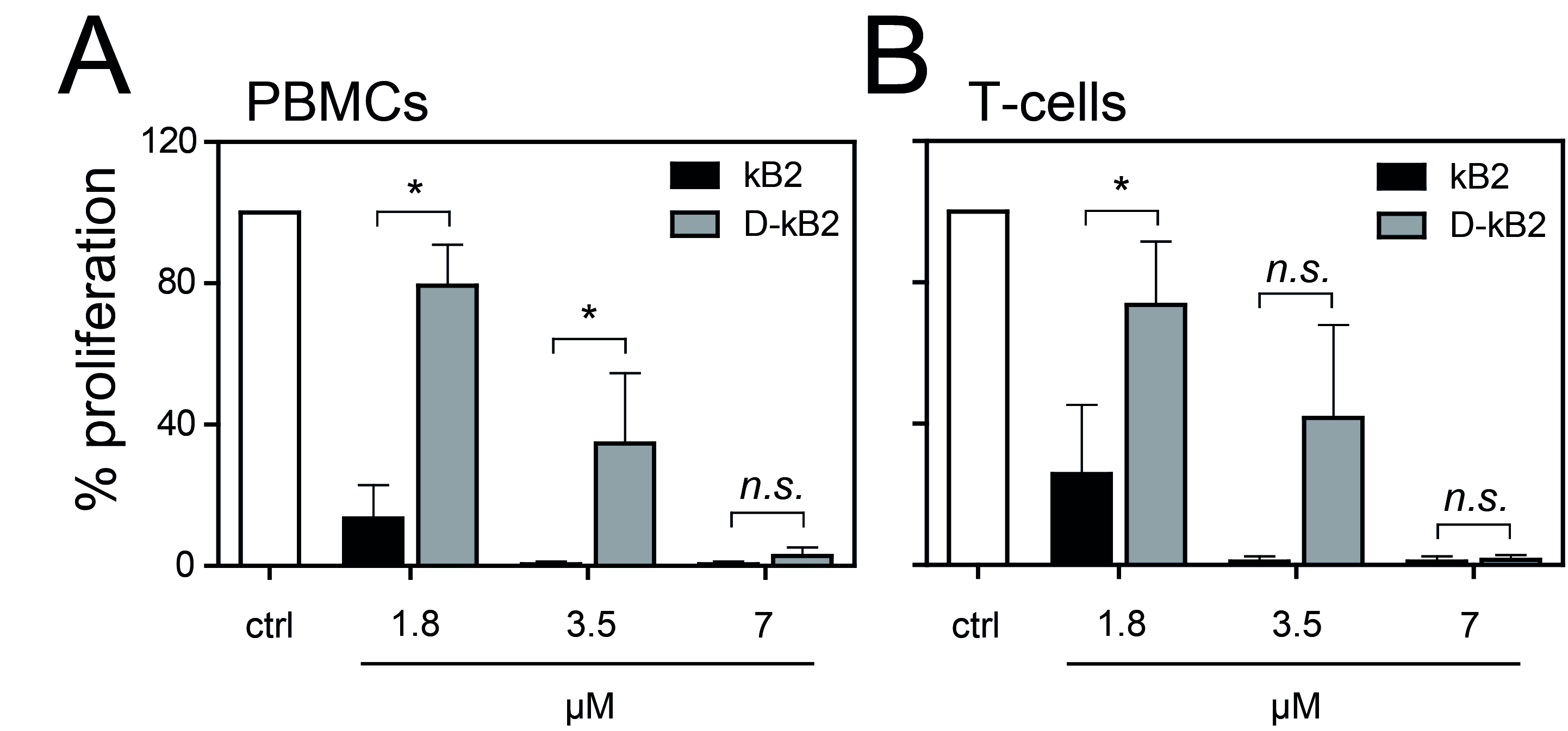

Supplement: Figure S3 — The influence of medium (ctrl), or kalata B2 and its all-D-enantiomer (D-kalata B2) at various concentrations (1.8-7 µM) on proliferation of activated primary lymphocytes (A) and purified T-cells (B) was measured by cell division analysis using CFSE-based flow cytometry on day three post stimulation. Data are presented as mean ± SD of three independent donors and experiments (*P <0.05; n.s. not significant). (TIF) [file pone.0068016.s003.tif]

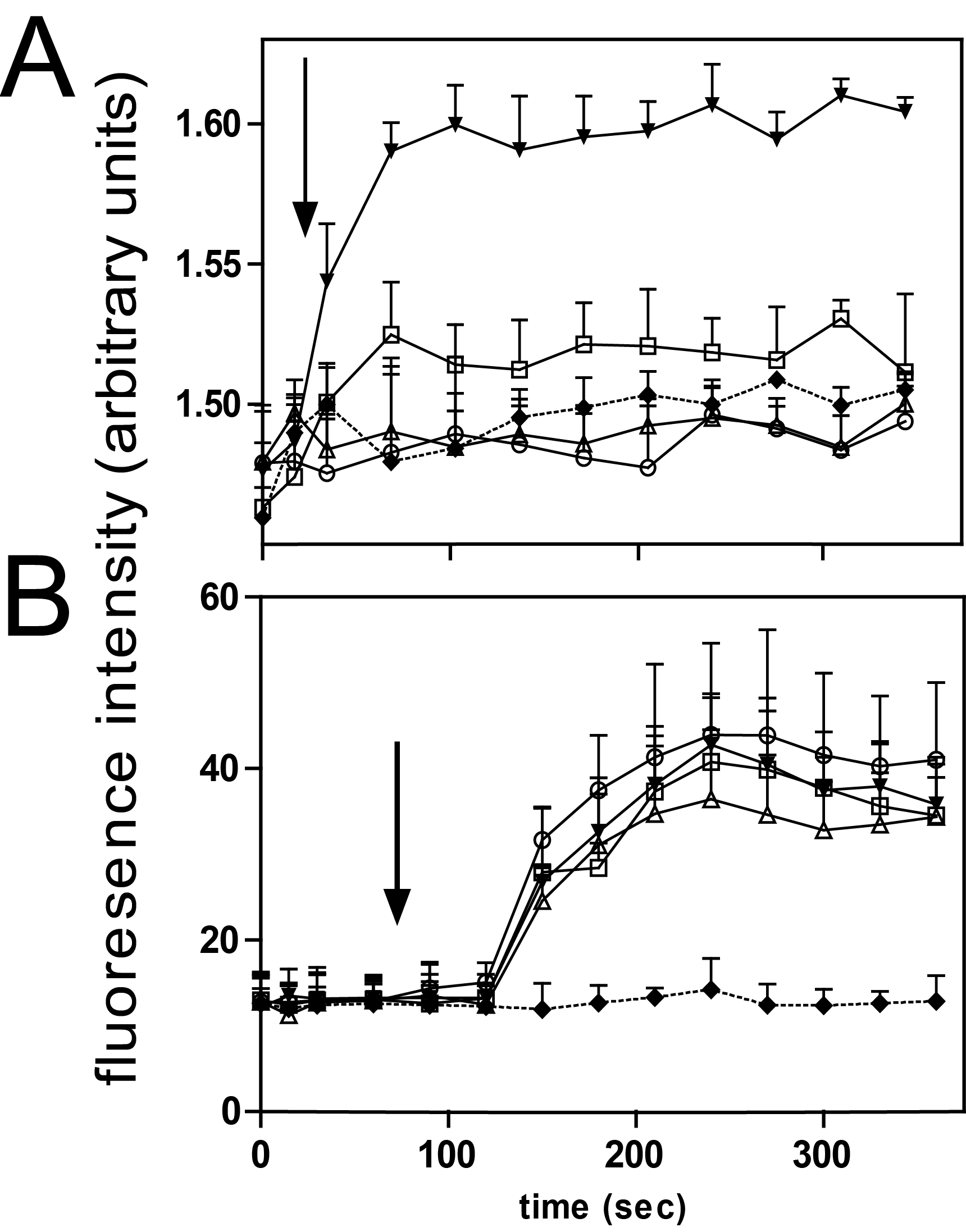

Supplement: Figure S4 — (A) The fluorescence time course of Fura-2 loaded human primary T-cells was measured by extinction at 340 and 380 nm, respectively and emission at 510 nm in 30 sec intervals, while continuously shaking. Ca2+-influx was initiated by adding compounds to the cells (illustrated by the arrow). To receive maximum Ca2+-release, cells were triggered with PMA (50 ng/mL) and ionomycin (500 ng/mL) (reversed triangles). For lowest Ca2+-levels, cells remained untreated (diamond-shapes, dotted line). Stimulation with [T20K] kalata B1 (open squares), [V10K] kalata B1 (open triangles) or cyclosporin A (open circles) (4 µM each) did not induce any change in Ca2+-signaling. (B) Ca2+-flux was additionally analyzed by FACS using Fluo-4 and Fura Red labeled T-cells, which were pretreated with cyclosporin A or cyclotides for 16 h. None of the compounds [T20K] kalata B1, [V10K] kalata B1 or cyclosporin A induced an inhibition of Ca2+-release after addition of PMA and ionomycin (indicated by the arrow). (TIF) [file pone.0068016.s004.tif]
